# Development of generalized Fourier and Fick’s law of electro-osmotic MHD flow of sodium alginate based Casson nanofluid through inclined microchannel: exact solution and entropy generation

**DOI:** 10.1038/s41598-022-21854-5

**Published:** 2022-11-04

**Authors:** Dolat Khan, Kanayo Kenneth Asogwa, Nevzat Akkurt, Poom Kumam, Wiboonsak Watthayu, Kanokwan Sitthithakerngkiet

**Affiliations:** 1 Fixed Point Research Laboratory, Fixed Point Theory and Applications Research Group, Center of Excellence in Theoretical and Computational Science (TaCS-CoE), Department of Mathematics, Faculty of Science, King Mongkut’s University of Technology Thonburi (KMUTT), 126 Pracha Uthit Rd., Bang Mod, Thung Khru, Bangkok, 10140 Thailand; 2 Department of Mathematics, Nigeria Maritime University, Okerenkoko, Delta State Nigeria; 3 Center of Excellence in Theoretical and Computational Science (TaCS-CoE), Faculty of Science, King Mongkut’s University of Technology Thonburi (KMUTT), 126 Pracha Uthit Rd., Bang Mod, Thung Khru, Bangkok, 10140 Thailand; 4 Department of Medical Research, China Medical University Hospital, China Medical University, Taichung, 40402 Taiwan; 5 Department of Mechanical Engineering, Munzur University, 62000 Tunceli, Turkey; 6 Intelligent and Nonlinear Dynamic Innovations Research Center, Department of Mathematics, Faculty of Applied Science, King Mongkut’s University of Technology North Bangkok (KMUTNB), 1518, Wongsawang, Bangsue, Bangkok, 10800 Thailand

**Keywords:** Applied mathematics, Nanoparticles

## Abstract

Electro-osmotic flow via a microchannel has numerous uses in the contemporary world, including in the biochemical and pharmaceutical industries. This research explores the electroosmotic flow of Casson-type nanofluid with Sodium Alginate nanoparticles through a vertically tilted microchannel. In addition, the transverse magnetic field is also considered. In this flowing fluid, the influence of heat and mass transmission is also explored. The aforementioned physical process is represented by partial differential equations. Utilizing suitable dimensionless variables for nondimensionalized. Furthermore, the non—dimensional classical system is fractionalized with the use of generalized Fourier and Fick's law. Generalizations are made using the Caputo derivative's description. The analytical solution of the velocity, temperature, and concentration profiles is obtained by combining the methods of Laplace and Fourier. Interestingly, the influence of several physical characteristics such as the fractional parameter, Casson fluid parameter, the thermal and mass Grashof numbers, and the zeta potential parameter is displayed. Moreover, the results show that the volume fractional of nanoparticles enhances the rate of heat transfer up to 39.90%, Skin friction up to 38.05%, and Sherwood number up to 11.11%. Also, the angle of inclination enhances the fluid velocity.

## Introduction

Fractional calculus advances the concept of classical calculus. Fractional calculus has proven to be an indispensable tool for mainstream science, as needful in optical fibers, electromagnetic, and plasma physics since it permits the formulation of differential equations that relate components and associated rates of growth. Owing to its inherited features and content memory repercussions, fractional operators have experienced a continual process of generalization and improvement over the past decades. Several fractional derivative applications exist in the fields of dynamics, chaos, chemical processes, rheological properties, and diffusion Hristov^1^. Incorporating the Caputo fractional (CF) operator with Fick's and Fourier's Laws led Khan et al.^2^ to the conclusion that the fractional technique is more realistic and useful than the traditional method since it provides a number of outputs that may improve the best fit of empirical data. Using Fourier's and Fick's generalized laws, Sheikh et al.^3^ looked into the flow of nanofluid. In their works, Rehman et al.^4^ and Ali et al.^5^ noted that conformable derivatives are used to approach the fractional differential equation. In light of a fractional-based model, Khan et al.^6^ came to the conclusion that the inclusion of hybrid nanoparticles rather than mono nanofluids controls the drilling fluid velocity. Moreover, the incorporation of different nanoparticle shapes into water increased the percentage of heat transfer by up to 11.149 percent. Jiang et al.^7^ used Fourier's and Fick's laws to assess the flow of nanofluid via a stretched surface. Through a comparison of fractional derivatives, Sheikh et al.^8^ investigated the flow of non-Newtonian fluids. Shah et al.^9^ study's of the erratic convective flow of viscous liquids using fractional Caputo-Fabrizio derivatives. Laplace transform combined with the Fourier-Sine transform was used by Shao et al.^10^ to analyze the MHD convective fluids. In a work employing the CF fractional derivative, Baleanu et al.^11^ conducted a mathematical investigation of the liver.

In 1959, The Casson fluid model was developed by Casson to describe the flow behavior of suspensions. Such liquids are classified as viscoelastic fluids, and they have numerous applications in the drilling process Asogwa et al.^12^. Many biomechanical and industrial applications exist for the Casson fluid model. For materials like blood, chocolate, and other rheological properties, the Casson fluid model is better than older viscoelastic models. Casson fluid is an endlessly viscous shear-thinning liquid with zero shear rate. On the other hand, a nanofluid is a solution containing nanoparticles that are predominantly metal or metal oxide with diameters ranging from 1 to 100 nm in a base fluid such as oil, water, or ethylene glycol. Nanofluids are favorable heat transfer fluids for engineering and manufacturing applications, according to data indicating the rapid expansion of nanofluids-related research. The evolution of heat transfer in nanofluids is mostly dependent on the heat conductivity of nanoparticles, particle volume concentration, and mass flow discharges. Using the Keller-box method, Rafique et al.^13^ explored the influences of Brownian motion on Casson nanofluids of boundary layer flow over an inclined stretched sheet, according to their findings, the improvement of Casson parameter slowed the velocity distribution and Nusselt number. Ghadikolaei et al.^14^ discussed the MHD Casson nanofluid flow over a sloped porous stretched sheet. Zeeshan et al.^15^ examined the MHD propagation of Casson Nanofluid across an enlarging permeable cylinder. They estimated that nanoparticle volume fraction dispersion decrease when the non-Newtonian fluid parameter improves, whereas friction increases by around 20 percent and the Sherwood number increases by about 0.5 percent. While the Nusselt number indicates a ten percent drop. Faisal et al.^16^ discussed the boundary layer flow of a Casson nanofluid driven by a moving surface. They found that for a given prescribed surface temperature, higher levels of the Casson parameter increase heat transport and deplete mass transfer. Khan et al.^17^ explored the viscoelastic fluid flow across a vertical channel. Using the analytical method, Khan et al.^18^ conducted a comparative investigation of Titanium oxide, Sodium alginate with Silver, Copper, and Aluminum oxide conveying nanofluid of MHD flow. Other relevant works are cited in^19–21^

Magnetohydrodynamics (MHD) refers to fluids that conduct electricity, such as plasma fluxes and liquid metals. Due to its prominent engineering applications, it has become a popular subject of research among scientists. Hydroelectric power plants, astronomy, geophysics, metallurgical industries, nuclear power plants, plasma propulsion in astronautics, and MHD generators, molecular and cellular biology, genetic engineering, medicine, and the development of delivery technologies are all areas where MHD may trace its ancestry^22,23^. The exact solution for the relative magnetic field was reported by Khan et al.^24^, for Casson fluid along with heat generation, chemical reaction, and Newtonian heating. Shah et al.^25^ reported the MHD free convection flow along with thermal memory. After that, the fractional analysis of the MHD flow of sodium alginate base fluid is reported by Khan et al.^26^, by implementing the definition of Atangana–Baleanu derivative of the non-local and non-singular kernel.

Due to the extensive medical applications of Electroosmosis and its aids in the treatment of diseases such as cellular abnormalities, sickle cells, and drug delivery employing diagnostic kits. The electroosmotic phenomena occur whenever a channel is packed with a liquid electrolyte and a sufficient voltage is applied, producing a charge on the inside wall of the container when the electrolyte gets into contact with the inner walls Haung et al.^27^. Arulanandam and Li^28^ explored microchannels using electroosmotic pumping. Chakraborty et al. ^29^ studied flow rate control in coupled pressure-driven microfluidic and electroosmotic processes. Yang et al.^30^ researched electroosmotic flow in microchannels, Horiuchi and Dutta^31^ evaluated electro osmotically driven microchannel flows. Using generalized Fick's and Fourier's law, Irshad et al.^32^ investigated the non-Newtonian fluid's electro-osmotic flow through a microchannel. They discovered that the velocity grows as the electrokinetic parameter values rise. This spike in velocity is attributed to the reason that a greater value of the electrokinetic parameter demonstrates an improvement in velocity. Hina et al.^33^ explored the electro-osmotic flow in a synchronous channel incorporating Carreau–Yasuda nanofluid They discovered that the quantity of confined boluses reduces as electro-osmotic force strength grows. In a simple microchannel, Zakeri^34^ evaluated electro-osmotic flow and polymerization characteristics. They revealed that increasing the electric field will enhance the mobility of the flexible polymer chain. Using the Debye-Hückel linearization, Oni and Jha^35^ examined the electroosmotic flow in a microchannel. The unsteady rotating electroosmotic flow of a paired stress fluid in a microchannel was studied by Siva et al.^36^ They discovered that as the couple stress characteristic rises, the axial electroosmotic flow velocity within the electrical double layer rises. Reddy et al.^37^ looked into the peristaltic Casson fluid motion caused by MHD electro-osmosis with energy transmission in a spinning asymmetric microchannel. They discovered that boosting electroosmotic force enhances the transport of heat rate.

From the perspective of the previously published work and considering the importance of non-Newtonian fluids and electro-osmotic flow in Engineering. The research aim of the recent study is to identify the behavior of the electroosmotic flow of Sodium Alginate Based Casson nanofluid fluid through a slanted vertical microchannel applying generalized Fick's and Fourier's laws. The model is developed in terms of PDEs for the selected flow pattern. The generated model is then fractionalized in a Caputo-derivative, and then employing Laplace and finite Fourier’s methods is used to provide results for energy, velocity, and concentration fields.

### Mathematical formulation

**Figure 1:**
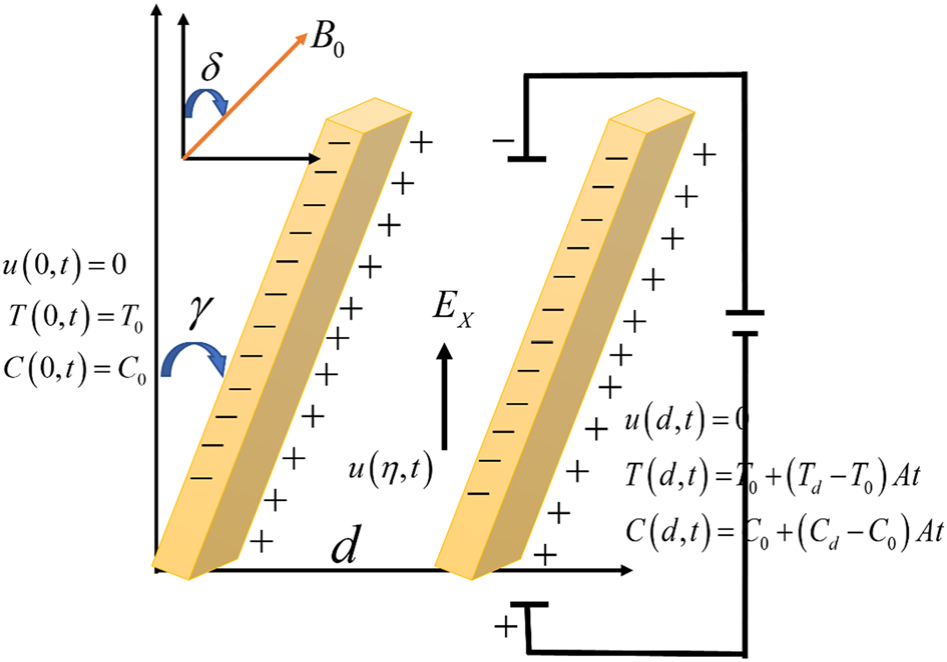
Geometry of the flow.

In this research, we study the electro-osmosis-induced unsteady MHD flow of the Sodium Alginate-based Casson fluid in a vertically inclined microchannel. The direction of the fluid flow is along x-axis. The flow of the fluid is in an inclined microchannel to maintain irregular zeta potential $\zeta_{1} ,\,\,\zeta_{2}$. The magnetic field $B_{0}$ is also applied to the fluid with the angle of inclination. Firstly, the fluid is at rest with ambient temperature $T_{0}$ and constant concentration $C_{0}$. After a period of time, the concentration and temperature are increased to $C_{0} + (C_{d} - C_{0} )At$ and $T_{0} + (T_{d} - T_{0} )At$ respectively as shown in Fig. 1.

Using the preceding assumptions, the mathematical form of the velocity, temperature, and concentration are given by^39,40^

1 $$\begin{aligned} \rho_{nf} \frac{{\partial u\left( {\eta ,t} \right)}}{\partial t} = & \mu_{nf} \left( {1 + \frac{1}{\beta }} \right)\frac{{\partial^{2} u\left( {\eta ,t} \right)}}{{\partial \eta^{2} }} - \sigma_{nf} B_{0}^{2} \sin (\delta )u\left( {\eta ,t} \right) + \left( {\rho \beta_{T} } \right)_{nf} g\cos (\gamma )\left( {T - T_{0} } \right) \\ & + \left( {\rho \beta_{C} } \right)_{nf} g\cos (\gamma )\left( {C - C_{0} } \right) + E_{X} \rho_{e} , \\ \end{aligned}$$

2 $$\frac{{\partial T\left( {\eta ,t} \right)}}{\partial t} = - \frac{1}{{\left( {\rho C_{p} } \right)_{nf} }}\frac{{\partial \overset{\lower0.5em\hbox{$\smash{\scriptscriptstyle\frown}$}}{q} \left( {\eta ,t} \right)}}{\partial \eta },$$

3 $$\overset{\lower0.5em\hbox{$\smash{\scriptscriptstyle\frown}$}}{q} \left( {\eta ,t} \right) = - k_{nf} \frac{{\partial T\left( {\eta ,t} \right)}}{\partial \eta },$$

4 $$\frac{{\partial C\left( {\eta ,t} \right)}}{\partial t} = - \frac{{\partial \overset{\lower0.5em\hbox{$\smash{\scriptscriptstyle\frown}$}}{j} \left( {\eta ,t} \right)}}{\partial \eta },$$

5 $$\frac{1}{{D_{nf} }}\overset{\lower0.5em\hbox{$\smash{\scriptscriptstyle\frown}$}}{j} \left( {\eta ,t} \right) = - \frac{{\partial C\left( {\eta ,t} \right)}}{\partial \eta },$$

6 $$\left. \begin{gathered} u\left( {\eta ,0} \right) = u\left( {0,t} \right) = u\left( {d,t} \right) = 0 \hfill \\ T\left( {\eta ,0} \right) = \,T\left( {0,t} \right) = T_{0} ,\,\,T\left( {d,t} \right) = \,T_{0} + \left( {T_{d} - T_{0} } \right)At, \hfill \\ C\left( {\eta ,0} \right) = C\left( {0,t} \right) = C_{0} ,\,\,\,C\left( {d,t} \right) = C_{0} + \left( {C_{d} - C_{0} } \right)At \hfill \\ \end{gathered} \right\}.$$

The dimensionless variables are:

7 $$\left. \begin{gathered} \,\,\tau = \frac{v}{{d^{2} }}t,\,\,\xi = \frac{\eta }{d},\,\,u^{ * } = \frac{u}{{u_{s} }},\,\varphi = \frac{{C - C_{0} }}{{C_{d} - C_{0} }},\,\theta = \frac{{T - T_{0} }}{{T_{d} - T_{0} }},\,k^{ * } = kd,\, \hfill \\ R_{\xi } = \frac{{\xi_{2} }}{{\xi_{1} }},\,\,\lambda = \frac{jd}{{D\left( {C_{d} - C_{0} } \right)}},\,\delta_{1} = \frac{qd}{{k\left( {T_{d} - T_{ \circ } } \right)}},\,\,\,A = \frac{v}{{d^{2} }} \hfill \\ \end{gathered} \right\}\,.$$

$$\left( {\rho C_{p} } \right)_{nf} = (1 - \phi )\left( {\rho C_{p} } \right)_{f} + \phi \left( {\rho C_{p} } \right)_{s} ,\,\,D_{nf} = \frac{{D_{f} }}{1 - \phi },\,\,\,\mu_{nf} = \frac{{\mu_{f} }}{{\left( {1 - \phi } \right)^{2.5} }},\,$$

$$\,k_{nf} = k_{f} \left( {\frac{{k_{s} + 2k_{f} - 2\phi (k_{f} - k_{s} )}}{{k_{s} + 2k_{f} + \phi \,(k_{f} - k_{s} )}}} \right),\;\left( {\rho \beta_{T} } \right)_{nf} = \left( {1 - \phi } \right)\,\left( {\rho \beta_{T} } \right)_{f} + \phi \left( {\rho \beta_{T} } \right)_{s} ,$$

$$\,\,\sigma_{nf} = \sigma_{f} \left( {1 + \frac{{3\left( {\sigma - 1} \right)\phi }}{{\left( {\sigma + 2} \right) - \left( {\sigma - 1} \right)\phi }}} \right)$$

8 $$\begin{aligned} \frac{{\partial u\left( {\xi ,\tau } \right)}}{\partial \tau } = & \frac{{m_{5} }}{{m_{4} }}\left( {1 + \frac{1}{\beta }} \right)\frac{{\partial^{2} u\left( {\xi ,\tau } \right)}}{{\partial \xi^{2} }} - M\frac{{m_{6} }}{{m_{4} }}\sin (\delta )u\left( {\xi ,\tau } \right) + Gr\frac{{m_{7} }}{{m_{4} }}\cos (\gamma )\theta \left( {\xi ,\tau } \right) \\ & + Gm\frac{{m_{8} }}{{m_{4} }}\cos (\gamma )\varphi \left( {\xi ,\tau } \right) + \frac{1}{{m_{4} }}k^{2} \left( {A_{1} e^{k\xi } + A_{2} e^{ - k\xi } } \right), \\ \end{aligned}$$

9 $$\frac{{\partial \theta \left( {\xi ,\tau } \right)}}{\partial \tau } = - \frac{{m_{3} }}{{m_{2} }}\frac{1}{\Pr }\frac{{\partial \delta \left( {\xi ,\tau } \right)}}{\partial \xi },$$

10 $$\delta_{1} \left( {\xi ,\tau } \right) = - \frac{{\partial \theta \left( {\xi ,\tau } \right)}}{\partial \xi },$$

11 $$\frac{{\partial \phi \left( {\xi ,\tau } \right)}}{\partial \tau } = - \frac{1}{{m_{1} }}\frac{1}{Sc}\frac{{\partial \lambda \left( {\xi ,\tau } \right)}}{\partial \xi },$$

12 $$\lambda \left( {\xi ,\tau } \right) = - \frac{{\partial \varphi \left( {\xi ,\tau } \right)}}{\partial \xi },$$

13 $$\left. \begin{gathered} u\left( {\xi ,0} \right) = u\left( {0,t} \right) = u\left( {1,t} \right) = 0 \hfill \\ T\left( {\xi ,0} \right) = \theta \left( {0,\tau } \right) = 0,\,\,\theta \left( {1,\tau } \right) = \tau \,, \hfill \\ \varphi \left( {\xi ,0} \right) = \varphi \left( {0,\tau } \right) = 0,\,\,\,\varphi \left( {0,\tau } \right) = \tau \hfill \\ \end{gathered} \right\},$$

where $M = \frac{{\sigma_{f} B_{ \circ }^{2} d^{2} }}{{\mu_{f} }}$ is Hartman number, $Gr = \frac{{gd^{2} \beta_{Tf} \left( {T_{d} - T_{o} } \right)}}{{v_{f} u_{S} }}$ for thermal Grashof number, $Sc = \frac{{v_{f} }}{{D_{f} }}$ represent the Schmidt number, $Gm = \frac{{gd^{2} \beta_{Cf} \left( {C_{d} - C_{o} } \right)}}{{v_{f} u_{S} }}$ represent mass Grashof number, $\Pr = \frac{{\mu_{f} c_{pf} }}{{k_{f} }}$ represent the Prandtl number, where $A_{1} = \frac{{R_{\xi } - e^{ - k} }}{2\sinh (k)},$ and $A_{2} = 1 - A_{1}$.

$$m_{1} = \frac{1}{1 - \phi },\,$$ $$m_{2} = (1 - \phi ) + \phi \frac{{\left( {\rho C_{p} } \right)_{s} }}{{\left( {\rho C_{p} } \right)_{f} }},\,\,\,$$ $$m_{3} = \frac{{k_{nf} }}{{k_{f} }},\,$$ $$m_{4} = 1 - \phi + \phi \frac{{\rho_{s} }}{{\rho_{f} }},\,\,$$ $$m_{5} = \frac{1}{{\left( {1 - \phi } \right)^{2.5} }},\,$$ $$m_{6} = 1 + \frac{{3\left( {\sigma - 1} \right)\phi }}{{\left( {\sigma + 2} \right) - \left( {\sigma - 1} \right)\phi }}$$, $$m_{7} = \left( {1 - \phi } \right)\, + \phi \frac{{\left( {\rho \beta_{T} } \right)_{s} }}{{\left( {\rho \beta_{T} } \right)_{f} }},\,\,$$ $$m_{8} = \left( {1 - \phi } \right)\, + \phi \frac{{\left( {\rho \beta_{C} } \right)_{s} }}{{\left( {\rho \beta_{C} } \right)_{f} }}$$.

### Generalized model

The simplified Fick's and Fourier law is utilized to fractionalize the model ^38,40^:

14 $$\frac{{\partial \theta \left( {\xi ,\tau } \right)}}{\partial \tau } = \frac{{m_{3} }}{{m_{2} }}\frac{1}{\Pr }{}^{c}D_{t}^{1 - \alpha } \frac{{\partial^{2} \theta \left( {\xi ,\tau } \right)}}{{\partial^{2} \xi }},$$

15 $$\frac{{\partial \varphi \left( {\xi ,\tau } \right)}}{\partial \tau } = \frac{1}{{m_{1} Sc}}{}^{c}D_{t}^{1 - \alpha } \frac{{\partial^{2} \varphi \left( {\xi ,\tau } \right)}}{{\partial \xi^{2} }},$$

where ${}^{c}D_{t}^{1 - \alpha }$ represent the time fractional Caputo operator.

To obtain the second equation in a more accurate form, take its inverse operator of fractional Caputo derivative we have:

16 $${}^{c}D_{t}^{\alpha } \theta \left( {\xi ,\tau } \right) = \frac{{m_{3} }}{{m_{2} }}\frac{1}{\Pr }\frac{{\partial^{2} \theta \left( {\xi ,\tau } \right)}}{{\partial^{2} \xi }},$$

17 $${}^{c}D_{t}^{\alpha } \varphi \left( {\xi ,\tau } \right) = \frac{1}{{m_{1} Sc}}\frac{{\partial^{2} \varphi \left( {\xi ,\tau } \right)}}{{\partial \xi^{2} }}.$$

### Problem solution

#### Energy field solution

By using transformation^38,40^

18 $$\chi_{1} \left( {\xi ,\tau } \right) = \theta \left( {\xi ,\tau } \right) - \xi f(\tau ).$$

Equation (16) becomes

19 $${}^{c}D_{t}^{\alpha } \chi_{1} \left( {\xi ,\tau } \right) + \xi {}^{c}D_{t}^{\alpha } f(\tau ) = \frac{{m_{3} }}{{m_{2} }}\frac{1}{\Pr }\frac{{\partial^{2} \chi_{1} \left( {\xi ,\tau } \right)}}{{\partial \xi^{2} }}.$$

The corresponding conditions are given by

20 $$\chi_{1} \left( {\xi ,0} \right) = 0,\,\chi_{1} \left( {0,\tau } \right) = 0,\,\chi_{1} \left( {1,\tau } \right) = 0.$$

Apply the joint Laplace and Fourier transform we get:

21 $$\overline{{\chi_{1\,F} }} \left( {n,q} \right) = \frac{{\left( { - 1} \right)^{n} }}{n\pi }\frac{{q^{\alpha - 2} }}{{q^{\alpha } + \frac{{m_{3} }}{{m_{2} }}\frac{{\left( {n\pi } \right)^{2} }}{\Pr }}}.$$

Taking inverse we get:

22 $$\chi_{1} \left( {\xi ,\tau } \right) = 2\sum\limits_{n = 1}^{\infty } {\frac{{\left( { - 1} \right)^{n} }}{\pi n}\sin \left( {\xi \pi n} \right)} \int\limits_{0}^{\tau } {E_{\alpha ,1} \left( {\frac{{m_{3} }}{{m_{2} }}\frac{{\left( {\pi n} \right)^{2} }}{\Pr }t^{\alpha } } \right)\left( {1 - t} \right)} \,dt.$$

The energy equation solution is as:

23 $$\theta \left( {\xi ,\tau } \right) = \chi_{1} \left( {\xi ,\tau } \right) + \xi f(\tau ).$$

### Concentration field solution

By using transformation^38,40^

24 $$\chi_{2} \left( {\xi ,\tau } \right) = \varphi \left( {\xi ,\tau } \right) - \xi g\left( \tau \right).$$

Equation (17) becomes

25 $${}^{c}D_{t}^{\alpha } \chi_{2} \left( {\xi ,\tau } \right) + \xi {}^{c}D_{t}^{\alpha } g\left( \tau \right) = \frac{1}{{m_{1} Sc}}\frac{{\partial^{2} \chi_{2} \left( {\xi ,\tau } \right)}}{{\partial \xi^{2} }},$$

Using boundary and initial conditions as we have

26 $$\chi_{2} \left( {\xi ,0} \right) = 0,\,\,\chi_{2} \left( {0,\tau } \right) = 0,\,\,\chi_{2} \left( {1,\tau } \right) = 0.$$

Using joint Laplace and sine finite Fourier transform we have:

27 $$\overline{{\chi_{2F} }} \left( {n,q} \right) = \frac{{\left( { - 1} \right)^{n} }}{n\pi }\frac{{q^{\alpha - 2} }}{{q^{\alpha } + \left( {\frac{{\left( {n\pi } \right)^{2} }}{{m_{1} Sc}}} \right)}}dt.$$

Taking inverse we get:

28 $$\chi_{2} \left( {\xi ,\tau } \right) = 2\sum\limits_{n = 1}^{\infty } {\frac{{\left( { - 1} \right)^{n} }}{\pi n}\sin \left( {\xi n\pi } \right)} \int\limits_{0}^{\tau } {E_{\alpha ,1} \left( {\frac{{\left( {\pi n} \right)^{2} }}{{m_{1} Sc}}t^{\alpha } } \right)\left( {1 - t} \right)\,} dt.$$

In last we get the equation of concentration as:

29 $$\varphi \left( {\xi ,\tau } \right) = \chi_{2} \left( {\xi ,\tau } \right) + \xi g(\tau ).$$

### Velocity profile solution

Using Laplace and Fourier transform as we have

30 $$\begin{aligned} \overline{{u_{F} }} \left( {n,q} \right) = & \frac{{\left( { - 1} \right)^{n + 1} H(q)}}{n\pi } + \left[ {\frac{{G_{3} }}{q} + \frac{{G_{2} }}{{q + G_{1} }}} \right]\frac{{( - 1)^{n} qH(q)}}{n\pi } + \frac{{Gr\,\frac{{m_{7} }}{{m_{4} }}\,\cos (\gamma )\,\overline{\theta }_{F} (n,q)}}{{q + G_{1} }} \\ & + \frac{{Gm\,\frac{{m_{8} }}{{m_{4} }}\,\cos (\gamma )\,\overline{\varphi }_{F} (n,q)}}{{q + G_{1} }} + \frac{1}{{q(q + G_{1} )}}\left[ {\frac{n\pi }{{\frac{1}{{m_{4} }}k^{2} + (n\pi )^{2} }}\left\{ \begin{gathered} A_{1} \left( {\left( { - 1} \right)^{n + 1} e^{k} + 1} \right) \hfill \\ + A_{2} \left( {\left( { - 1} \right)^{n + 1} e^{ - k} + 1} \right) \hfill \\ \end{gathered} \right\}} \right], \\ \end{aligned}$$

where

$$\begin{aligned} G_{0} = & \frac{{m_{5} }}{{m_{4} }}\left( {\frac{\beta + 1}{\beta }} \right), \\ G_{1} = & \frac{{m_{6} }}{{m_{4} }}\sin (\delta )M + G_{0} (\pi n)^{2} , \\ G_{2} = & G_{0} (n\pi )^{2} , \\ G_{3} = & 1 - G_{1}^{ - 1} G_{0} (n\pi )^{2} . \\ \end{aligned}$$

Taking inverse we get:

31 $$\begin{aligned} u\left( {\xi ,\tau } \right) = & 2\sum\limits_{n = 1}^{\infty } {\left[ {\frac{{\left( { - 1} \right)^{n} }}{\pi n}H(\tau )*\left( {G_{2} e^{{ - G_{1} \tau }} + G_{3} H(\tau )} \right)} \right]} \sin \left( {\xi n\pi } \right) \\ & + 2Gr\frac{{m_{7} }}{{m_{4} }}\cos (\gamma )\sum\limits_{n = 1}^{\infty } {\left[ {e^{{ - G_{1} \tau }} \frac{{\left( { - 1} \right)^{n + 1} }}{\pi n} * \left( {\int\limits_{0}^{\tau } {E_{\alpha ,1} \left( {\frac{{m_{3} }}{{m_{2} }}\frac{{ - \left( {\pi n} \right)^{2} }}{\Pr }t^{\alpha } } \right)\left( {t - \tau } \right)\,} dt} \right)} \right]} \sin \left( {\xi n\pi } \right) \\ & + 2\tau \xi + 2Gm\frac{{m_{8} }}{{m_{4} }}\cos (\gamma )\sum\limits_{n = 1}^{\infty } {\left[ {e^{{ - G_{1} \tau }} \frac{{\left( { - 1} \right)^{n + 1} }}{\pi n} * \left( {\int\limits_{0}^{\tau } {E_{\alpha ,1} \left( {\frac{{ - \left( {\pi n} \right)^{2} }}{{m_{1} Sc}}t^{\alpha } } \right)\left( {t - \tau } \right)\,} dt} \right)} \right]} \sin \left( {\xi n\pi } \right) \\ & + 2\sum\limits_{n = 1}^{\infty } {\left[ {\left( {\frac{{1 - e^{{ - G_{1} \tau }} }}{{G_{1} }}} \right)\left( {\frac{\pi n}{{\frac{1}{{m_{4} }}k^{2} + (\pi n)^{2} }}\left\{ {A_{1} \left( { - 1} \right)^{n + 1} e^{k} + A_{2} \left( { - 1} \right)^{n + 1} e^{ - k} + A_{2} + A_{1} } \right\}} \right)} \right]} \sin \left( {\xi n\pi } \right), \\ \end{aligned}$$

where $E_{\alpha ,1}$ and $H(\tau )$ are Mittag Leffler and unit step function.

### Nusselt and sherwood number

Nusselt and Sherwood are dimensionless numbers. Mathematically it can be written as:

32 $$Nu = \left. { - \frac{{k_{nf} }}{{k_{f} }}\frac{{\partial \theta \left( {\xi ,\tau } \right)}}{\partial \xi }} \right|_{\xi = 1}$$

33 $$Sh = D_{nf} \left. {\frac{{\partial \varphi \left( {\xi ,\tau } \right)}}{\partial \xi }} \right|_{\xi = 1}$$

### Skin friction

Mathematically Skin friction for the left place can be written as:

34 $$S_{f} = m_{5} \left( {1 + \frac{1}{\beta }} \right)\left. {\frac{{\partial u\left( {\xi ,\tau } \right)}}{\partial \xi }} \right|_{\xi = 0}$$

Similarly, skin friction in right place can be written as:

35 $$S_{f} = \left( {1 + \frac{1}{\beta }} \right)\left. {\frac{{\partial u\left( {\xi ,\tau } \right)}}{\partial \xi }} \right|_{\xi = 1}$$

### Entropy generation

Entropy production for the flow of a Casson nano fluid in the presence of a magnetic field and without a mass concentration is described by^42,43^, taking into consideration the velocity from Eq. (23) and (31).

36 $$Ns\left( {\xi ,\tau } \right) = m_{3} \left( {\frac{{\partial \theta \left( {\xi ,\tau } \right)}}{\partial \xi }} \right)^{2} + m_{6} \frac{{MB_{r} }}{\Omega }\left( {u\left( {\xi ,\tau } \right)} \right)^{2} + m_{5} \frac{{B_{r} }}{\Omega }\left( {\frac{{\partial u\left( {\xi ,\tau } \right)}}{\partial \xi }} \right)^{2},$$

where $\Omega$ and $B_{r}$ is the dimensionless temperature difference and Brinkman number, denoted by,

$$B_{r} = \frac{\mu }{{\kappa \left( {T_{W} - T_{\infty } } \right)}},\,\,\Omega = \frac{{T_{W} - T_{\infty } }}{{T_{\infty } }}.$$

Additionally, the Bejan number $B_{e}$ is known as,

37 $$B_{e} = \frac{{N_{H} \left( {\xi ,\tau } \right)}}{{Ns\left( {\xi ,\tau } \right)}},$$

where $N_{H} \left( {\xi ,\tau } \right) = m_{3} \left( {\frac{{\partial \theta \left( {\xi ,\tau } \right)}}{\partial \xi }} \right)^{2}$ is the entropy generation due to heat transfer.

The Bejan number is well recognized as a useful tool for describing how fluid fraction and magnetic field regulate thermal transfer.

## Results and discussion

**Figure 2:**
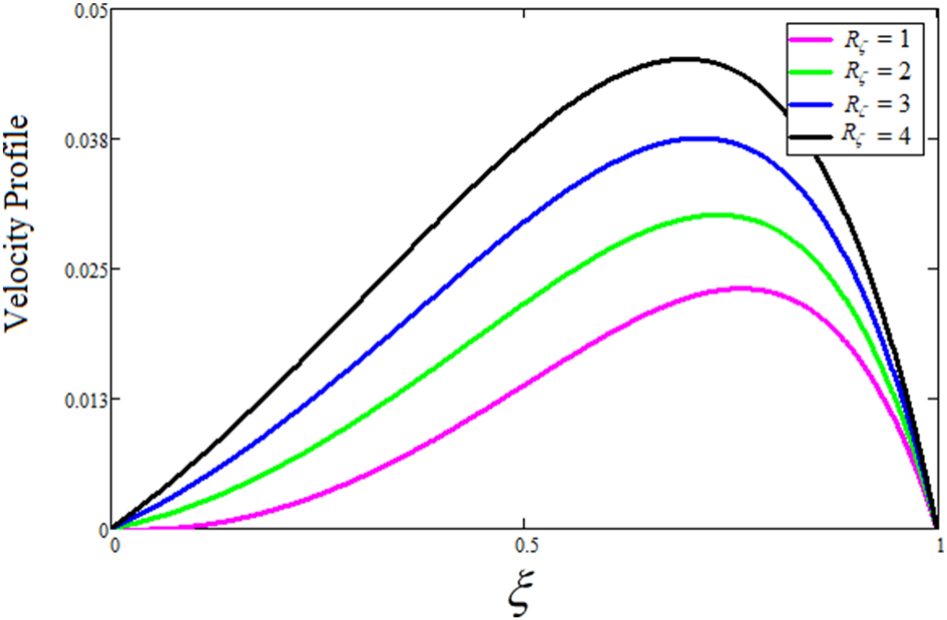
The consequence of $R_{\zeta }$ on the velocity distribution. where $\phi = 0.04,\delta = \gamma = \frac{\pi }{3},M = 5,k = 0.3,Gr = 10,Gm = 20,\beta = 0.2,\alpha = 0.5,t = 1,Sc = 10.$

The velocity, energy, and concentration profiles of sodium alginate-based Casson nanofluid through an inclined microchannel, are examined in this section along with the effects of other factors. The results of this investigation are reported in Figs. 2 through 20.

**Figure 3:**
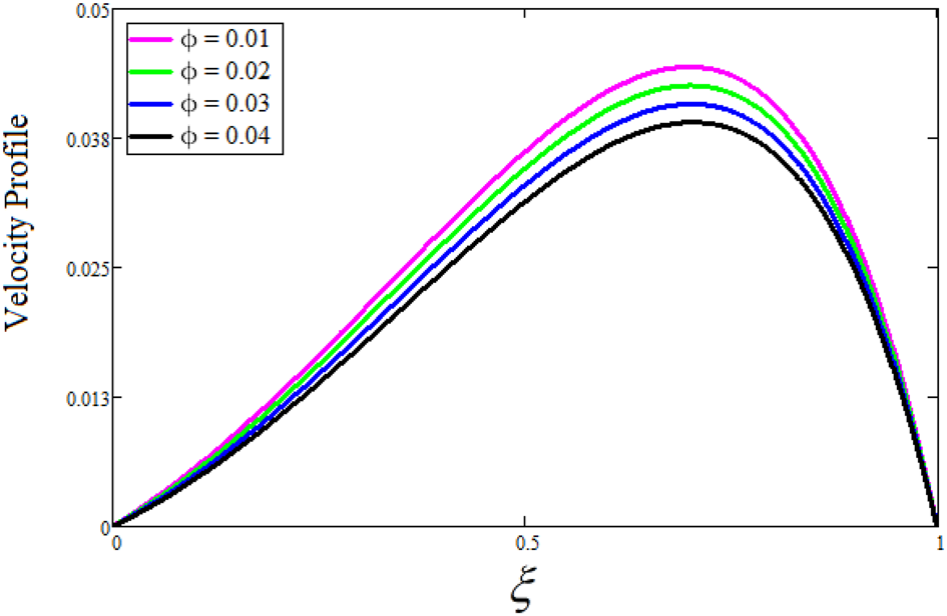
The consequence of $\phi$ on the velocity distribution. Where $R_{\zeta } = 2,\delta = \gamma = \frac{\pi }{3},M = 5,k = 0.3,Gr = 10,Gm = 20,\beta = 0.2,\alpha = 0.5,t = 1,Sc = 10,$

**Figure 4:**
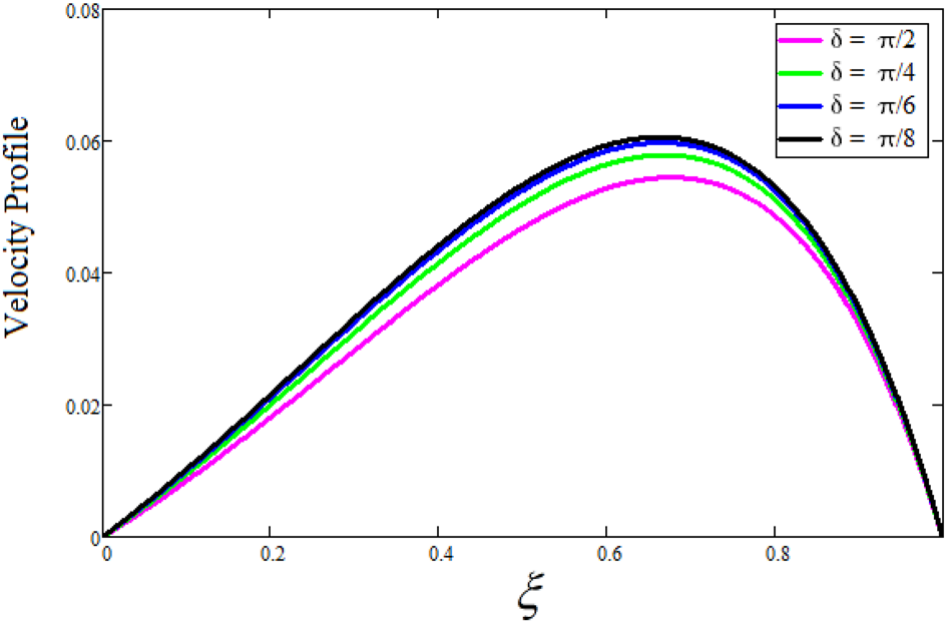
The effect of $\delta$ (inclined angle of magnetic field) on velocity of fluid. Where $R_{\zeta } = 2,\phi = 0.04,\gamma = \frac{\pi }{3},M = 5,k = 0.3,Gr = 10,Gm = 20,\beta = 0.2,\alpha = 0.5,t = 1,Sc = 10,$

**Figure 5:**
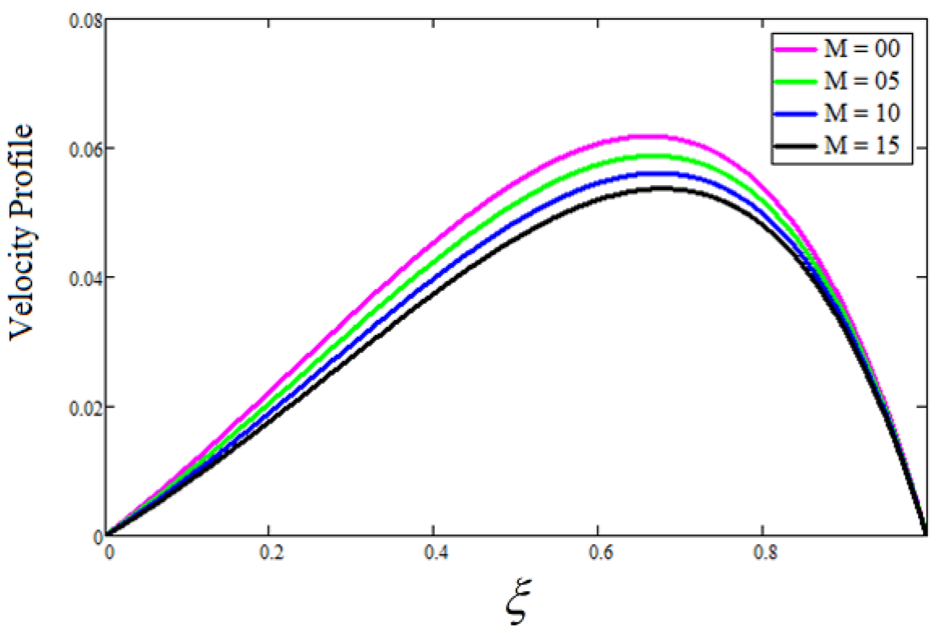
The consequence of $M$ on velocity of fluid. Where $R_{\zeta } = 2,\phi = 0.04,\delta = \gamma = \frac{\pi }{3},k = 0.3,Gr = 10,Gm = 20,\beta = 0.2,\alpha = 0.5,t = 1,Sc = 10,$

**Figure 6:**
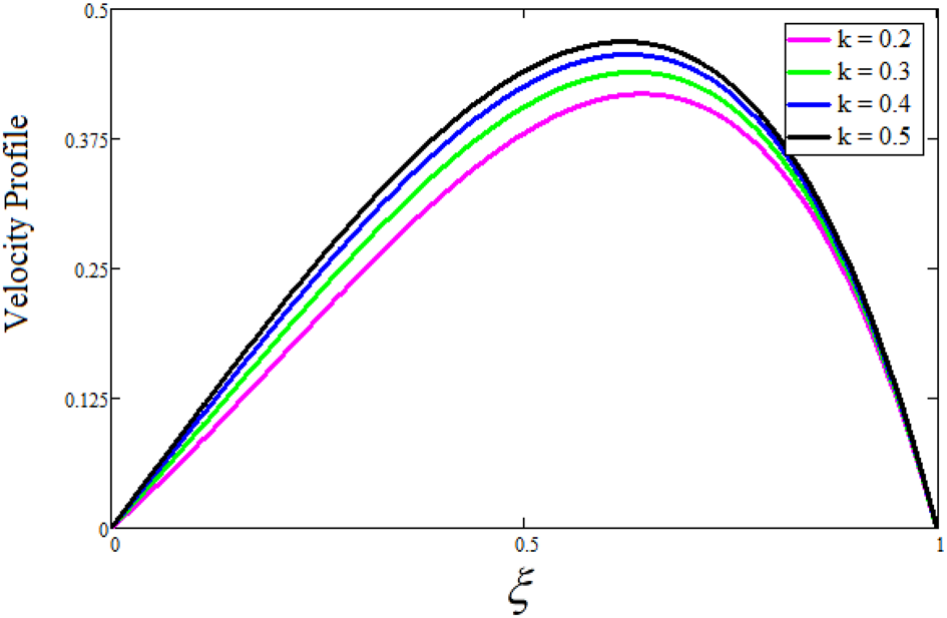
The consequence of $k$ on the velocity distribution. Where $R_{\zeta } = 2,\phi = 0.04,\delta = \gamma = \frac{\pi }{3},M = 5,Gr = 10,Gm = 20,\beta = 0.2,\alpha = 0.5,t = 1,Sc = 10,$

**Figure 7:**
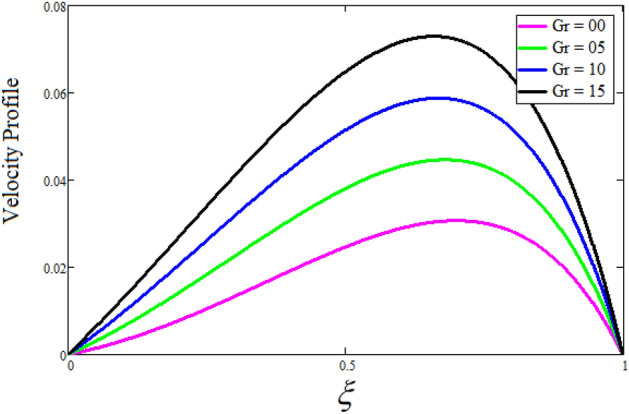
The consequence of $Gr$ on the velocity distribution. Where $R_{\zeta } = 2,\phi = 0.04,\delta = \gamma = \frac{\pi }{3},M = 5,k = 0.3,Gm = 20,\beta = 0.2,\alpha = 0.5,t = 1,Sc = 10,$

**Figure 8:**
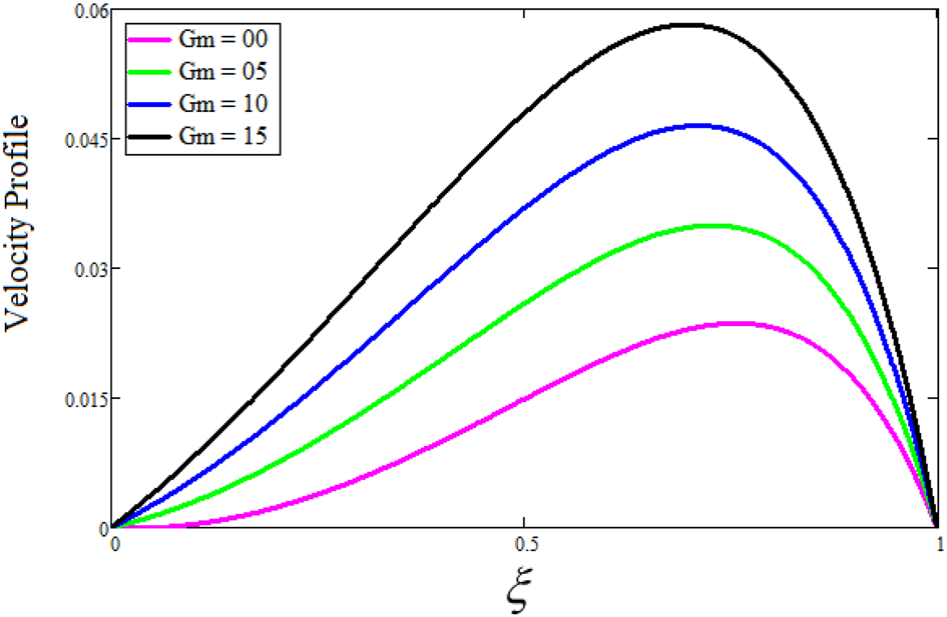
The consequence of $Gm$ impact on the velocity distribution. Where $R_{\zeta } = 2,\phi = 0.04,\delta = \gamma = \frac{\pi }{3},M = 5,k = 0.3,Gr = 10,\beta = 0.2,\alpha = 0.5,t = 1,Sc = 10,$

**Figure 9:**
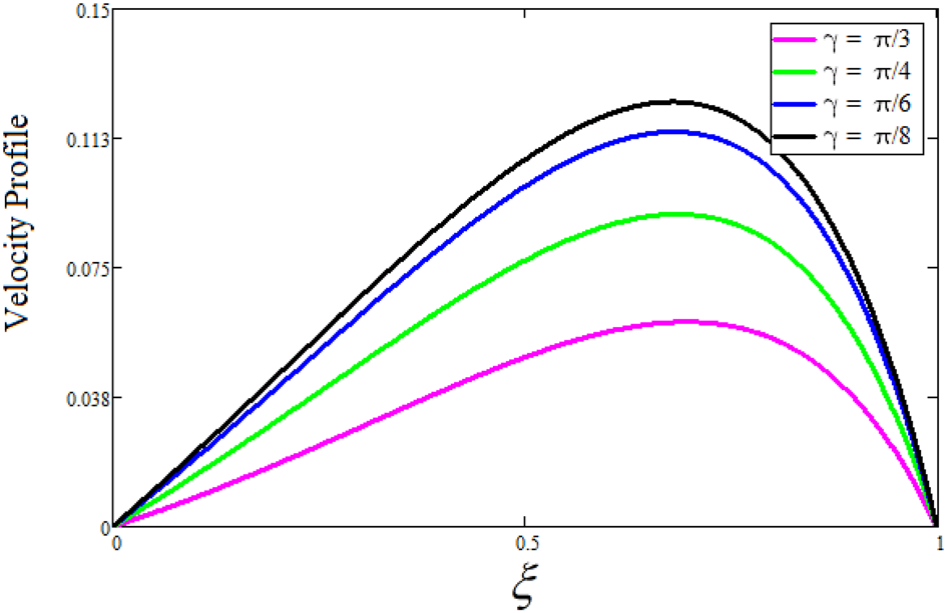
The consequence of angle of plate inclination on velocity distribution. Where $R_{\zeta } = 2,\phi = 0.04,\delta = \frac{\pi }{3},M = 5,k = 0.3,Gr = 10,Gm = 20,\beta = 0.2,\alpha = 0.5,t = 1,Sc = 10,$

**Figure 10:**
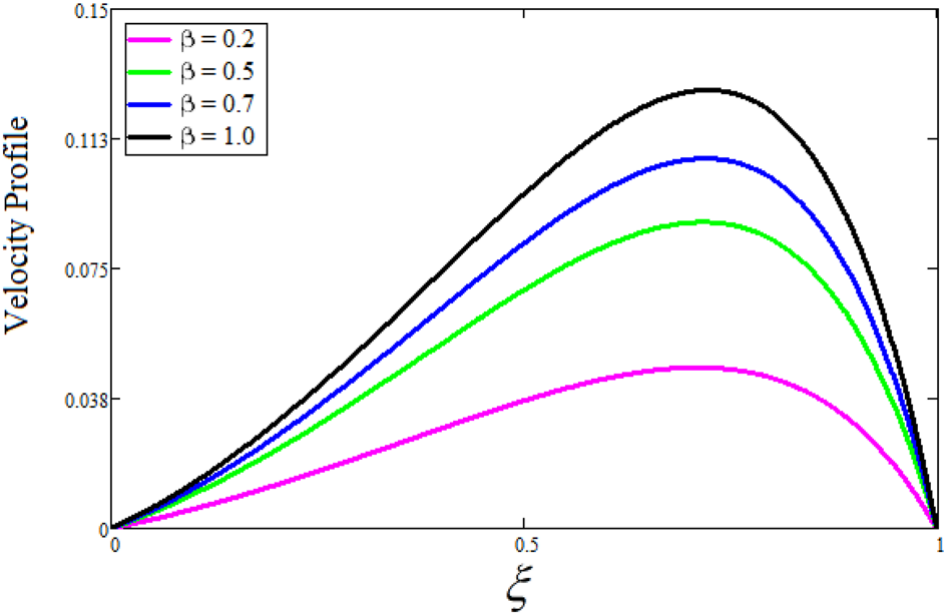
The consequence of $\beta$ on velocity distribution. Where $R_{\zeta } = 2,\phi = 0.04,\delta = \gamma = \frac{\pi }{3},M = 5,k = 0.3,Gr = 10,Gm = 20,\alpha = 0.5,t = 1,Sc = 10,$

**Figure 11:**
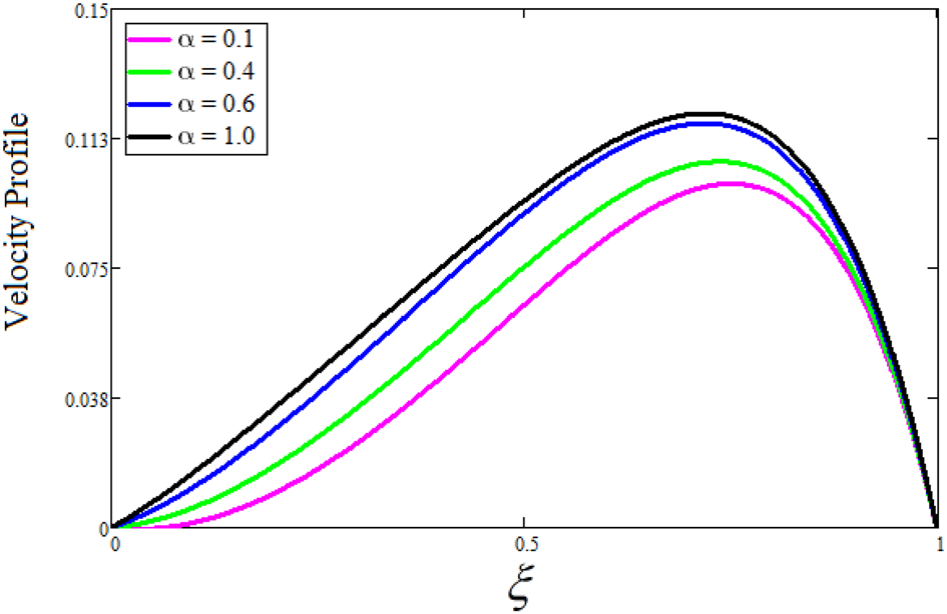
The consequence of $\alpha$ on velocity distribution. Where $R_{\zeta } = 2,\phi = 0.04,\delta = \gamma = \frac{\pi }{3},M = 5,k = 0.3,Gr = 10,Gm = 20,\beta = 0.2,t = 1,Sc = 10,$

**Figure 12:**
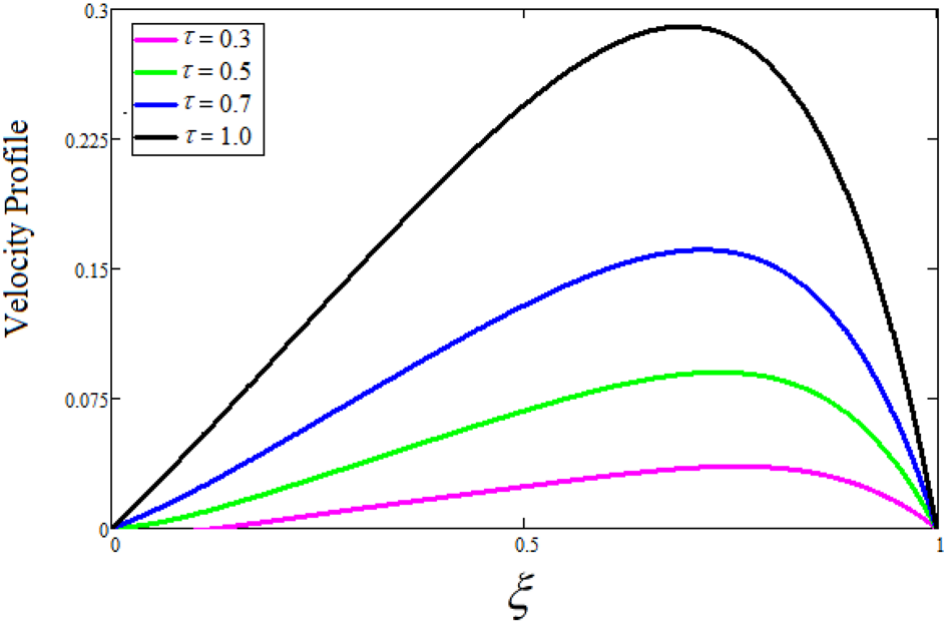
The consequence of $t$ on velocity distribution. Where $R_{\zeta } = 2,\phi = 0.04,\delta = \gamma = \frac{\pi }{3},M = 5,k = 0.3,Gr = 10,Gm = 20,\beta = 0.2,\alpha = 0.5,Sc = 10,$

**Figure 13:**
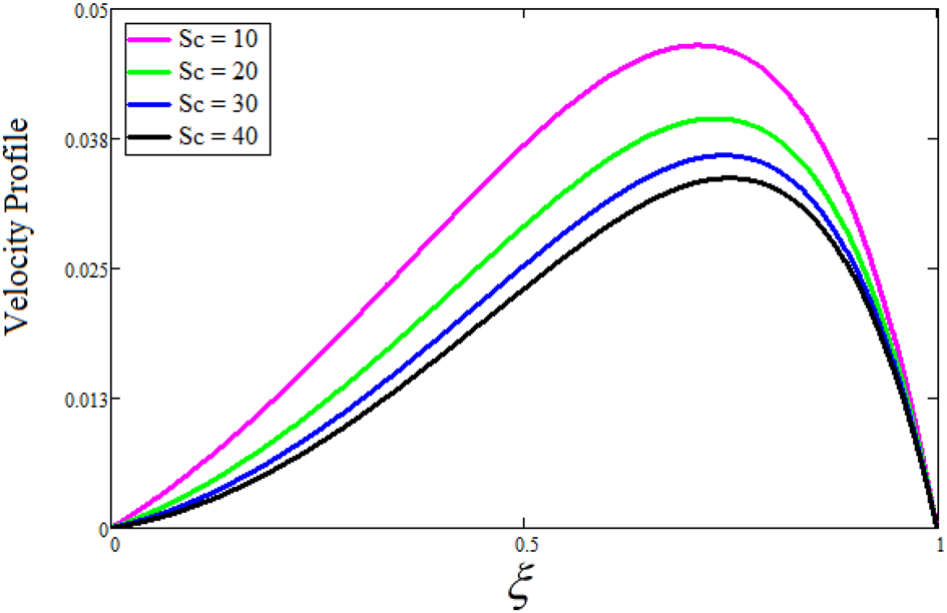
The consequence of $Sc$ on the velocity distribution. Where $R_{\zeta } = 2,\phi = 0.04,\delta = \gamma = \frac{\pi }{3},M = 5,k = 0.3,Gr = 10,Gm = 20,\beta = 0.2,\alpha = 0.5,t = 1.$

**Figure 14:**
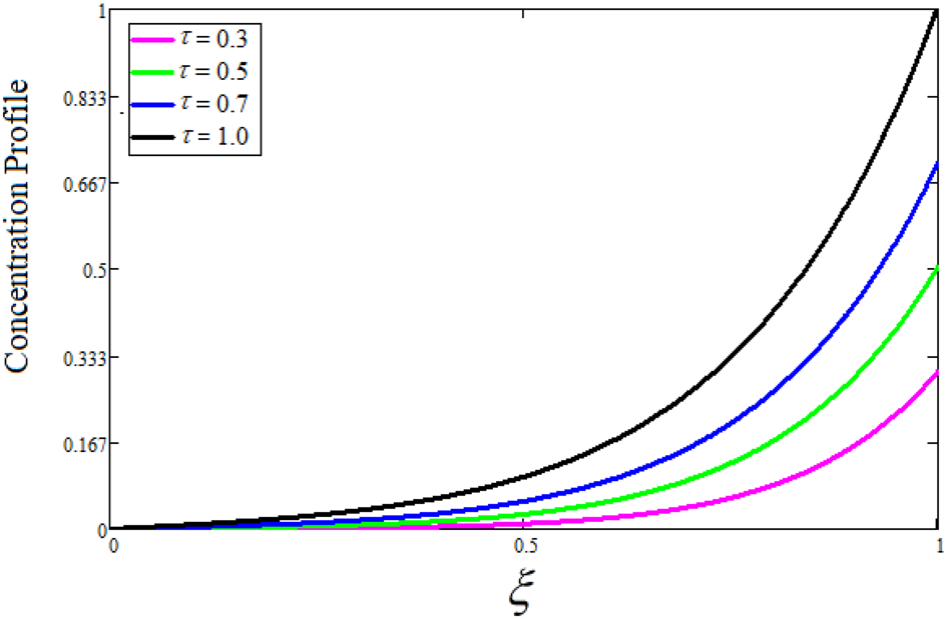
The consequence of $t$ on concentration distribution. Where $\phi = 0.01,\alpha = 0.5,Sc = 15.$

**Figure 15:**
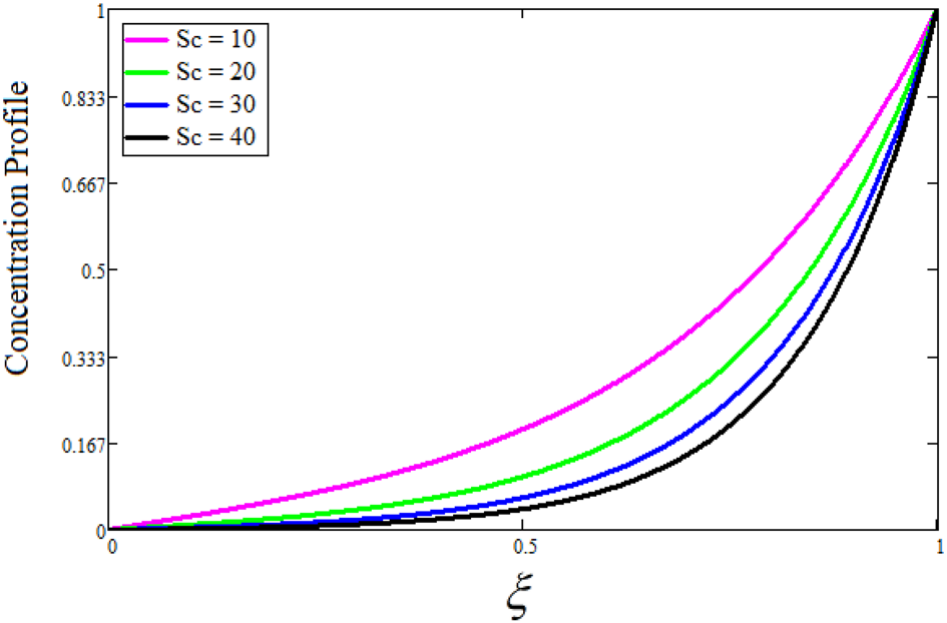
The consequence of $Sc$ on the concentration distribution. Where $\phi = 0.01,\alpha = 0.5,t = 1.$

**Figure 16:**
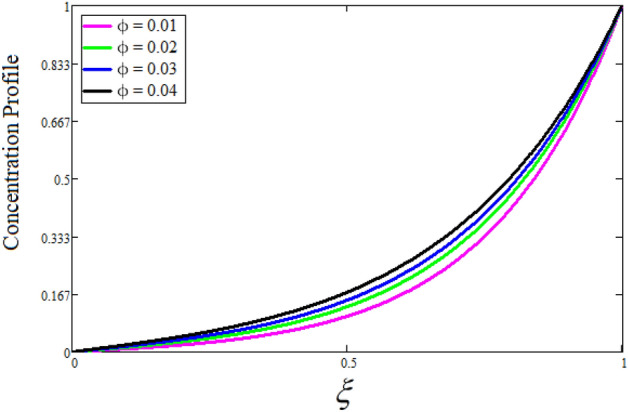
The consequence of $\phi$ on concentration distribution. Where $\alpha = 0.5,t = 1,Sc = 15.$

**Figure 17:**
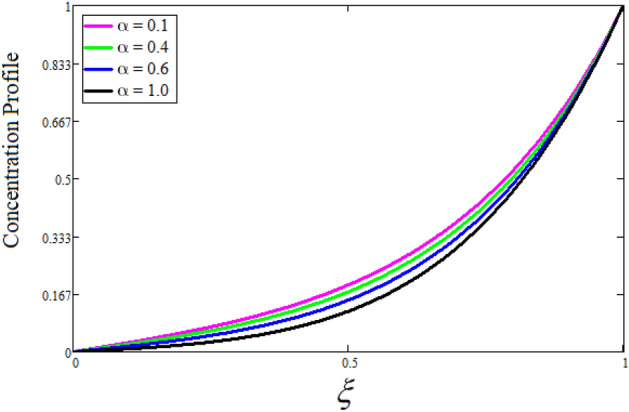
The consequence of $\alpha$ on concentration distribution. Where $\phi = 0.01,t = 1,Sc = 15.$

**Figure 18:**
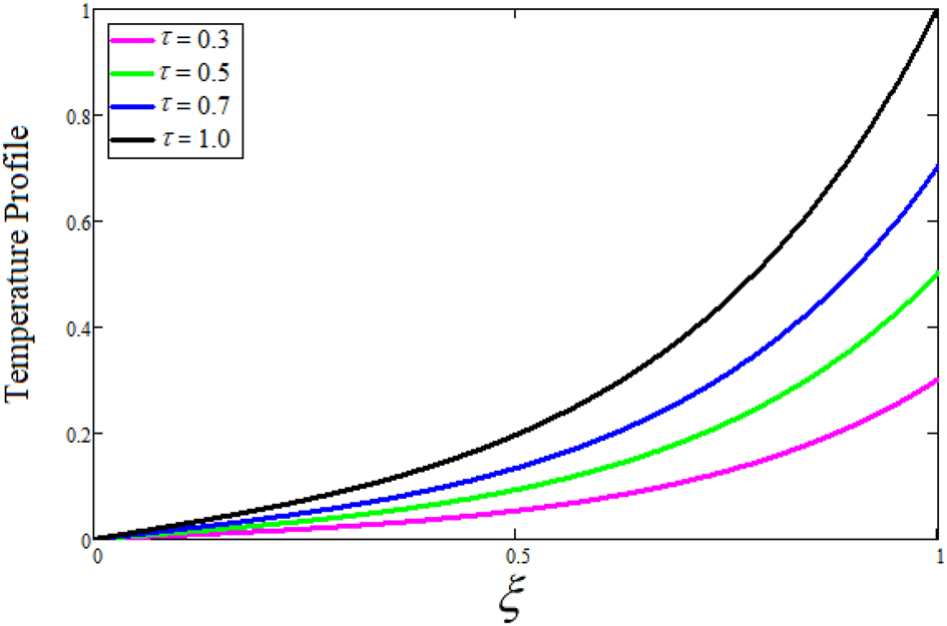
The consequence of $t$ on temperature distribution. Where $\phi = 0.01,\alpha = 0.5.$

**Figure 19:**
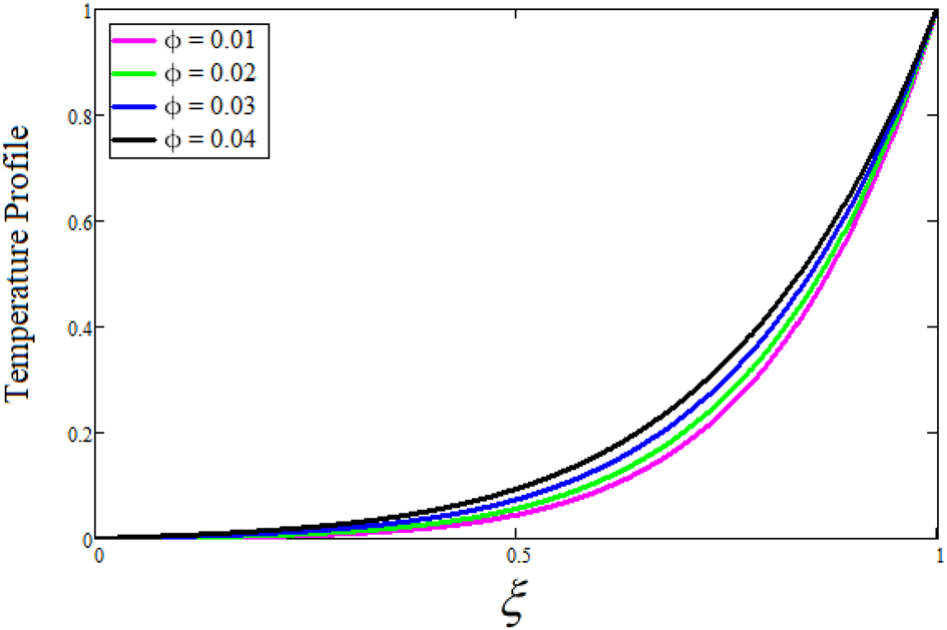
The consequence of $\phi$ on temperature distribution. Where $\alpha = 0.5,t = 1.$

**Figure 20:**
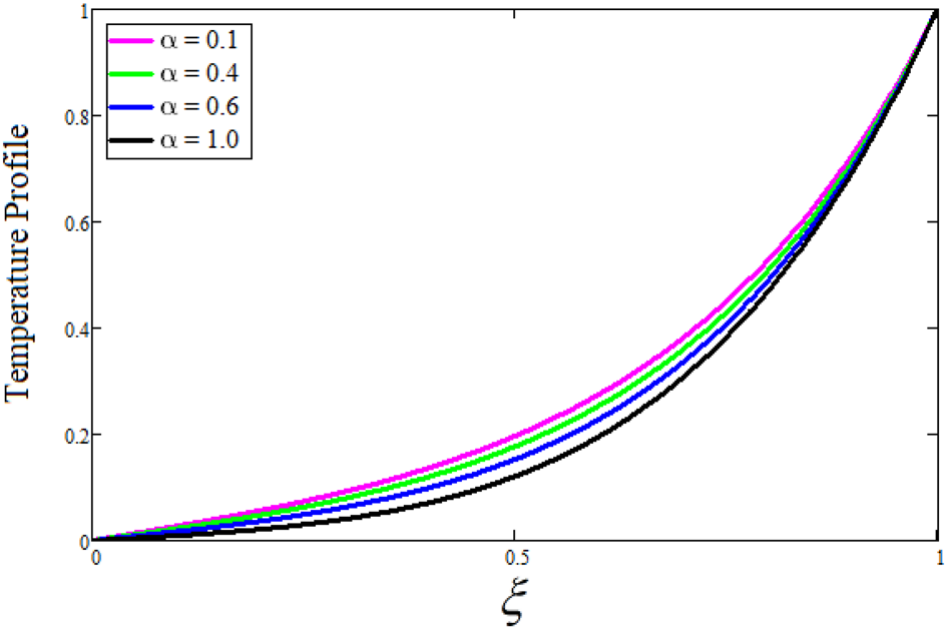
The consequence of $\alpha$ on temperature distribution. Where $\phi = 0.01,t = 1.$

**Figure 21:**
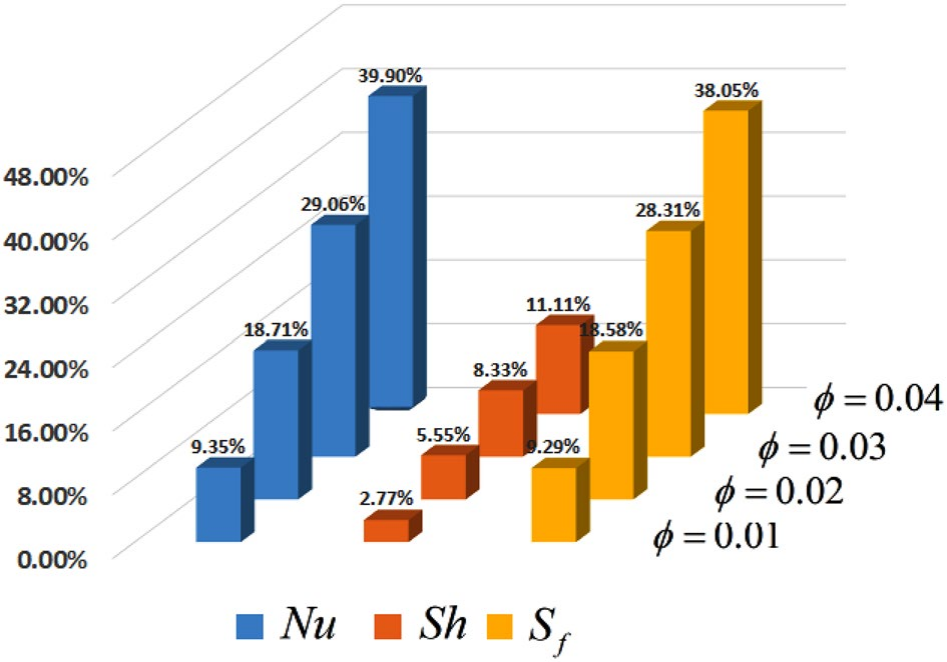
Percentage of $Nu,\,\,Sh$ and $S_{f}$ against volume fractional of nanofluid.

**Figure 22:**
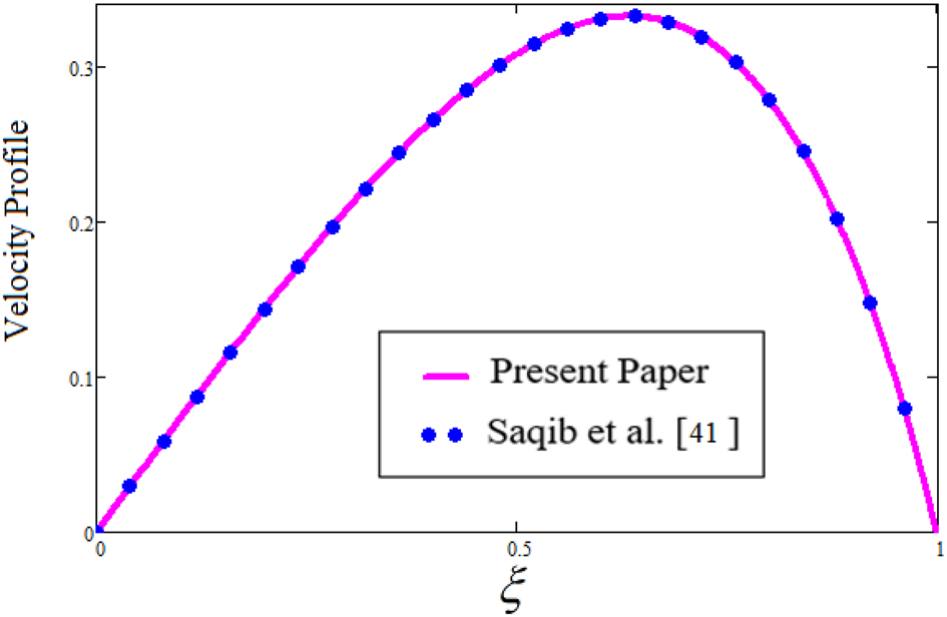
Comparison study with Saqib et al*.*^41^.

**Figure 23:**
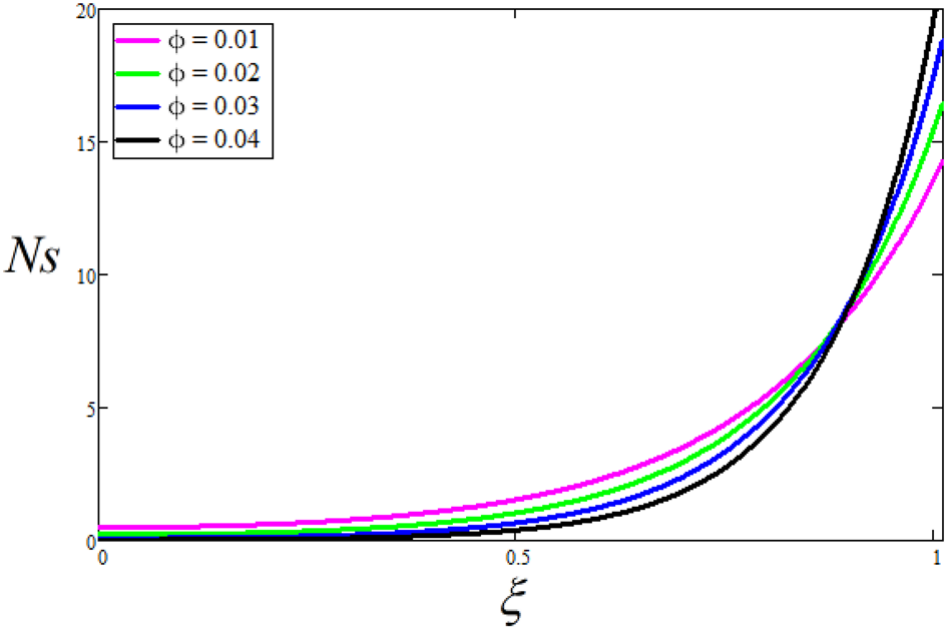
The consequence of $\phi$ on the Entropy generation. Where $R_{\zeta } = 2,\delta = \gamma = \frac{\pi }{3},M = 5,k = 0.3,Gr = 10,Gm = 20,\beta = 0.2,\alpha = 0.5,t = 1,Sc = 10,B_{r} = 0.5,\Omega = 5.$

**Figure 24:**
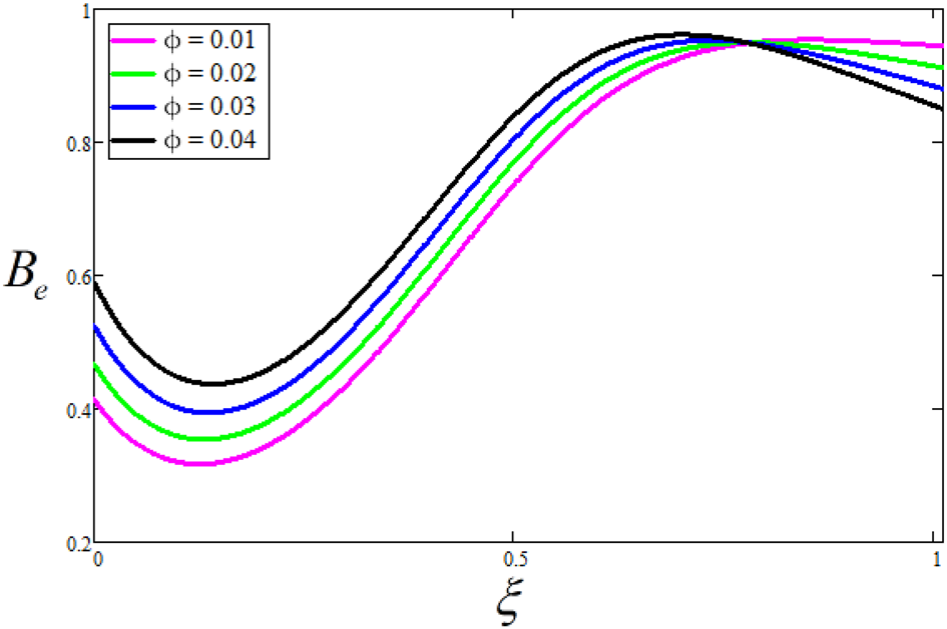
The consequence of $\phi$ on the Bejan number. Where $R_{\zeta } = 2,\delta = \gamma = \frac{\pi }{3},M = 5,k = 0.3,Gr = 10,Gm = 20,\beta = 0.2,\alpha = 0.5,t = 1,Sc = 10,B_{r} = 0.5,\Omega = 5.$

Figure 2 depicts the effect of the Zeta potential on the velocity profile. The heightening values of Zeta potential improve the velocity of the nanofluid, Physically, The stability of colloidal nanoparticles is significantly and easily quantified by the zeta potential. The size of the zeta potential represents the strength of electrostatic attraction between nearby nanoparticles with identical charges in a dispersion. Consequently, the velocity profile is enhanced. Figure 3 depicts the effect of the volume fraction of Sodium Alginate on the velocity profile. The stronger the volume fraction of the nanofluid, the slower the velocity of the nanofluid, which indicates that the nanoparticles are physically impeding the flow of the fluid. It is due to the fact that increasing the concentration of nanoparticles in the Sodium alginate base fluid developed the resistive forces which make them slow down in the channel. The higher the concentration in the fluid increases the viscosity of the Sodium alginate as a result the fluid velocity declines. An increase in the inclined angle of the magnetic field on the velocity profile induces an upshot of the velocity profile, as can be seen in Fig. 4. Physically, the velocity of the fluid is slowed because the Lorentz forces increase with increasing the angle of magnetic field inclination. Figure 5 displays the impact of the magnetic field ($$M$$) on the velocity distribution from 0 to 15. The magnetic field counters the velocity of nanofluid transmission. In principle, a rise in the magnetic field causes a substantial decrease in non—dimensional velocity. This is because the magnetic field creates a body force described as the Lorentz force, which retards motion. Figure 6 illustrates the behavior of electro-kinetic separation characteristic (k) on the velocity field under the impact of $$k$$ values between 0.2 and 0.5. It is detected that the fluid velocity climbs as $$k$$ increases. The thinner Electric Double Layer (EDL) that results from a higher value of k increases the electro-osmotic flow's effectiveness in a region with a bigger velocity gradient, leading to a greater overall velocity. The EDL is a collection of ions that accumulates on the vertical channel's two parallel plates. Figure 7 presents the implication of $$Gr$$ on the velocity distribution. the heightening values of $$Gr$$ from 0 to 15 captured in Fig. 7 on the velocity profile lead to an escalation of the nanofluid's velocity. Physically, When $$Gr$$ increases, buoyancy forces rise, causing a drop in the thickness of the momentum boundary layer, which causes a robust improvement of velocity distribution. Figure 8 depicts the impact of $$Gm$$ on the velocity profile. which shows a spike in the velocity profile as the values of $$Gm$$ growing. This occurred as a result of the gradient of concentration, which contributed to the enhanced buoyancy forces in the nanofluid, leading to an increase in the velocity profile. The influence of the angle of plate inclination on the velocity of the nanofluid is shown to be represented in Fig. 9, and it is detected that the velocity profile of the nanofluid is a feature that increases as the angle of plate inclination increases. Physically, the angle of plate inclination is enhancing the fraction due to which retardation occurs in fluid velocity. Figure 10 depicts the fluctuations in velocity profile owing to changes in Casson fluid parameter. From this figure, intriguing conclusions can be drawn. The velocity increases as the Casson parameter value goes up. However, it can be asserted that a Casson fluid with a greater value will respond like a Newtonian fluid. The significance of the fractional parameter on the velocity profile is depicted in Fig. 11. The higher fractional parameter values on the velocity profile give more velocity of the fluid while other parameters are fixed. Physically, the variation of fractional parameters reports the more realistic results for the given physical model. Which is best for experimentalist to compare their results. Figure 12 is projected to represent the influence of time on the nanofluid velocity and is seen that the velocity of the nanofluid is an escalating function of time. It is due to the fact that the fluid model is unsteady so it must depend on time. Figure 13 exhibits the dynamics of the nanofluid velocity profile under the influence of $$Sc$$ values between 10 and 40. As $$Sc$$ rises, it can be seen that the fluid velocity decreases. This is the case because when $$Sc$$ grows, the nanofluid's viscosity improves, the mass diffusion rate lowers, and the velocity drops. The effect that time has on the concentration of nanofluid is illustrated in Fig. 14, and it can be examined that the concentration of nanofluid increases with time. In Fig. 15, which depicts the effect of the Schmidt number on the nanofluid concentration profile, it is seen that nanofluid mass concentration drops as the $$Sc$$ increases. The molecular diffusion coefficient to kinematic viscosity ratio is known as the Schmidt number. As shown in Fig. 16, the increment of nanoparticle volume fraction of Sodium Alginate is responsible for the improvement of nanofluid mass concentration caused by colloidal forces. Figure 17 highlights the relevance of the fractional parameter on the temperature profile. The elevated fractional parameter values on the nanofluid deplete the temperature profile. Figure 18 indicates the impact of time on the temperature of the nanofluid, and it can be observed that the temperature of the nanofluid increases as time passes. The interaction between the nanoparticle volume fraction of Sodium Alginate and the temperature profile is shown in Fig. 19. Heat transmission rises as the volume percentage of nanoparticles in Sodium Alginate grows. Figure 20 provides a visual representation of the impact that the fractional parameter on the concentration profile. The lower concentration profile is caused by the larger fractional parameter values of the nanofluid. Figure 21 highlighted the influence of volume fractional of nanofluid against Nusselt number, Sherwood number and skin fraction. It is examined that the enhancement of volume fractional of nanofluid enhances the Nusselt number, Sherwood number and skin fraction up to 39.90%, 11.11% and 38.05% respectively. By putting $$R_{\zeta } = 0,\phi = 0,\delta = \gamma = \frac{\pi }{2},M = 1.5,k = 0,Gr = 5,$$$$Sc = 0,$$$$Gm = 0,\alpha = 0.5,$$ and $$\beta \to \infty ,$$ our solution is reduced to the solution of Saqib et al.^41^, which is presented in Fig. 22. Figures 23 and 24 presents the consequence of nanoparticle volume fraction on entropy generation and Bejan number respectively. The variation of nanoparticle volume enhances the fluid more denser which caused the retardation of entropy generation and as a results the Bejan number enhance.

## Conclusion

In this study, we explored the electroosmotic flow conveying Sodium Alginate non-Newtonian nanofluid via an inclined vertical microchannel utilizing generalized Fourier and Fick's law and engaging Laplace and Fourier's techniques. The following are the most important findings from the study:

- The energy pattern of Sodium Alginate nanofluid rises with increasing nanoparticle volume fraction and duration, with a diminishing contribution as the fractional parameter increases.
- The concentration of nanofluid for Sodium Alginate enhances with the developing quantities of nanoparticle volume fraction and time with a drop consequence as a fractional parameter and Schmidt number enlarge.
- The heightening values of Zeta potential, electro-kinetic separation, thermal and mass Grashof numbers, angle of plate inclination, Casson fluid, fractional parameter, and time boost the velocity of the nanofluid for Sodium Alginate, whereas the magnetic field, nanoparticle volume fraction, and Schmidt number constitute opposite effect.
- The enhancement of volume fractional of nanofluid enhances the Nusselt number, Sherwood number and skin fraction up to 39.90%, 11.11% and 38.05% respectively.
- The volume fractional of nanoparticle retard the entropy generation while enhance the Bejan number.

## Data Availability

Data of this study will be made available from the corresponding author on reasonable request.

## List of symbols

$\rho_{nf}$: Density of nanofluid
$\mu_{nf}$: Dynamic viscosity of nanofluid
$\sigma_{nf}$: Electrical conductivity of nanofluid
$$B_{0}^{{}}$$: Magnetic flux
$$g$$: Gravitational acceleration
$$T_{0}$$: Amended temperature
$\delta ,\gamma$: Angle of inclinations
$$C_{0}$$: Amended concentration
$$E_{X}$$: Electric field
$$C_{p}$$: Heat capacitance
$D$: Coefficient of diffusion
$Gm$: Mass Grashof number
$Gr$: Thermal Grashof number
$$\Pr$$: Prandtl number
$u\left( {\eta ,t} \right)$: Velocity of the fluid
$$\beta$$: Casson fluid
$u_{s}$: Helmholtz-smoluchowski velocity
$\beta_{T}$: Coefficient of the thermal expansion
$$T$$: Temperature profile
$$\beta_{C}$$: Concentration coefficient
$$C$$: Concentration Profile
$R_{\xi }$: Ratio of zeta potential
$\rho_{e}$: Net charge density
$$k$$: Thermal conductivity
$\phi$: Volume fraction of nanofluid
$$Sc$$: Schmidt number
$$M$$: Magnetic parameter
